# Kentucky State Medical Society

**Published:** 1854-04

**Authors:** 


### KENTUCKY STATE MEDICAL SOCIETY.

In our last number we alluded briefly to the third meeting of the Kentucky Medical Society, held at Lexington, in October, and to the fact of a deficiency in its revenues. The Secretary has furnished us with some memoranda of the meeting, and addressed to the members of the Society a circular letter, which we publish in the hope that it may cause the treasury to be replenished.

At the Lexington meeting, which took place on the 19th of October, the following reports were read;—on the *Medical Literature of Kentucky* by Dr. L. P. Yandell; on *Improvements in Surgery*, by Dr. J. B. Flint; on the *Relations of Geology and Disease*, by Dr. Robert Peter; on the *Statistics of Hernia*, by Dr. S. B. Richardson; on the *History and Mode of Management of Prisons and Penitentaries*, by Dr. W. C. Sneed; on *Medical Ethics*, by Dr. R. C. Hewitt; on *Public Hygiene*, by Dr. T. S. Bell; on *Medical Grievances in Courts af Justice*, by Dr. H. Wible; on *Epidemic Erysipelas*, by Dr. W. T. Owen; on *Vital Statistics*, by Dr. W. L. Sutton.

Objections were made to the report of Dr. Flint, on the ground that he had availed himself of the opportunity afforded by an appointment of the Society to assail certain members to whom he was unfriendly, and on motion of Dr. J. M. Duke, of Maysville, the Committee of Publication was instructed to strike out all that part of the report which related to medical ethics. This led Dr. Flint to ask permission of the Society to withdraw his report, which was granted; but at a subsequent period of the meeting, at the suggestion of the President, Dr. Gross, the vote on the resolution of Dr. Duke was reconsidered, and it was voted to request Dr. Flint to hand his paper to the publishing committee.

On the first evening of the meeting an appropriate address was delivered by Dr. W. S. CHIPLEY, the retiring President, to an audience of gentlemen and ladies. The tone, style, and sentiments of the address were worthy of the highest officer of the Kentucky Medical Society.

Dr. G. W. BAYLESS, of Cincinnati, and Dr. H. KIRBY, of Mississippi, were invited to take seats in the Society and to participate in its discussions.

Dr. A. CLAPP, of New Albany, Dr. J. F. SMITH, of New York, and Dr. M. L. LINTON, of St. Louis, were elected Honorary members of the Society.

The following gentlemen were elected officers of the Society for the ensuing year:

Dr. S. D. GROSS, of Louisville, *President.*
Dr. AYRES, of Lexington, *Senior Vice President.*
Dr. CHAMBERS, of Covington, *Junior Vice President.*
Dr. W. C. SNEED, of Frankfort, *Recording Secretary.*
Dr. W. H. MILLER, of Louisville, *Corresponding Secretary.*
Dr. MONROE, of Frankfort, *Librarian.*
Dr. D. D. THOMSON, of Louisville, *Treasurer.*

The following gentlemen compose the Committee of Publication:—Dr. T. G. Richardson, Dr. T. S. Bell, and Dr. John Bartlett.

The following gentlemen were appointed delegates to the American Medical Association, which meets on the 1st of May, in St. Louis:—Drs. Anderson, Chew, Thomson, Evans, Pilkington, Peter, Duke, Bradford, Ewen, Mattingly, Miller, Chipley, and Darby.

Several amendments to the By-Laws of the Society were adopted, among which are the following:—that the annual meetings of the Society shall be opened with prayer; that new members shall pay the initiation fee before being entitled to vote; and that no member shall be entitled to vote who has not paid the assessment which is to be fixed at each annual meeting.

The Chairmen of the various Standing and Special Committees for the ensuing year are as follows:

STANDING COMMITTEES.

*Arrangements.*—Dr. Preston, of Covington.
*Practical Medicine.*—Dr. Chew, of Woodford.

*Improvements in Pharmacy.*—Dr. J. L. Smith, of Louisville.
*Vital Statistics.*—Dr. John Swain.
*Obstetrics.*—Dr. L. Powell, of Louisville.
*Medical Ethics.*—Dr. Mattingly, of Bardstown.
*Public Hygeine.*—Dr. P. B. Drake.
*Epidemics.*—Dr. H. M. Bullitt, of Louisville.
*Surgery.*—Dr. F. Polin, of Springfield.
*Indigenous Botany.*—Dr. Donohoff, of Louisville.
*Finances.*—Dr. Hewitt, of Louisville.

---

### SPECIAL COMMITTEES.

*Medical Biography.*—Dr. R. J. Breckinridge, Louisville.
*Suits for Malpractice.*—Dr. Spillman, Harrodsburg.
*Relation between Disease and Particular Geological Formations.*—Dr. Peter, Lexington.
*Statistics of Lithotomy and Calculous Diseases.*—Dr. Gross, Lousville.
*History and Mode of Management of Hospitals.*—Dr. Raphael, Louisville.
*Results of Surgical Operations in Malignant Diseases.*—Dr. T. W. Colescott, Louisville.
*Placenta Previa.*—Dr. H. Miller, Louisville.
*Inflammation and Ulceration of Cervix Uteri.*—Dr. W. H. Miller, Louisville.
*Epidemic Dysentery.*—Dr. Hynes, Bardstown.
*Typhoid Fever.*—Dr. J. Smith, Danville.
*Statistics of Remedies in Disease.*—Dr. L. Rogers, Louisville.
*Scarlatina.*—Dr. Evans, Mercer county.
*Ovariotomy.*—Dr. Bradford.
*Comparative value of Remedies in Tubercular Disease.*—Dr. Craig.
*Substitutes for Quinine.*—Dr. Chambers, Covington.
*Spinal Disease.*—Dr. Freeman.
*Insanity.*—Dr. J. R. Allen, Lexington.
*Physiological and Morbid Effects of Alcoholic Drinks.*—Dr. J. M. Duke, Maysville.
*Injuries of the Skull and Brain.*—Dr. Hardin, Louisville.
*Therepeutical Effects of Anthemis Cotula in Disease.*—Dr. N. B. Anderson, Louisville.
*Water as a Thereaputic Agent.*—Dr. Darby, Lexington.

Among numerous resolutions passed, the one relating to the publication of the transactions of the society, seems likely to remain a dead letter. On motion of Dr. Spillman, the Society resolved, that in future no Chairman of a Special Committee shall occupy more than an hour and a half in reading his report; and on motion of Dr. Chambers it was resolved, that the Recording Secretary be directed to notify delinquent members that their names would be erased from the books of the Society, unless they paid their dues regularly. The following resolutions were also adopted:

*Whereas* the benefit resulting to the public, directly and indirectly, from the action and unwearied labors of the medical profession, are so numerous and important, that physicians are entitled to the utmost consideration and respect from the public; they ought to entertain a just appreciation of professional qualifications; to make a proper distinction between true science, and the assumptions of ignorance and empiricism; to afford every facility and encouragement for the acquisition of medical education, and instead of allowing the Statute Books to exhibit the anomaly of exacting knowledge from physicians under liabilities to heavy penalties for resorting to the means of obtaining it, they are entitled to the confidence, and ought to receive the patronage of the State; Therefore be it *Resolved*, That a Committee of five be appointed whose duty it shall be to memorialize the next General Assembly in reference to the passage of a law authorizing the publication of the Transactions of the Kentucky State Medical Society, and that 500 copies be furnished annually to the President for gratuitous distribution.

*Resolved*, That the Secretary, at an early day, as practicable, cause to be printed 150 copies of the above preamble and resolutions, and send one to each Senator and Representative elect in the Commonwealth.

Drs. Chew, H. Miller, Sutton and Mattingly were appointed the committee.

*Resolved*, That a Committee be appointed by this Society whose duty it shall be to memorialize the next session of the Legislature of Kentucky, to pass a law, making it obligatory on apothecaries and druggists, and all venders of medicine of every description to place a label on every article of patent medicines and nostrums, which label shall have written or printed upon it, the name of every article which enters into its composition, and the quantity of each, written in plain English.

Drs. Chambers, Drake and Small were appointed under this resolution.

On motion of Dr. Sutton it was *resolved*, That the nomenclature of diseases proposed by the American Medical Association be adopted by this Society.

The Society instructed the publishing committee to have printed 300 extra copies of Dr. Sutton's report on Vital Statistics, and to request the author to furnish an abstract for the newspapers of the State.

A sumptuous and elegant entertainment was provided for the Society by the Physicians of Lexington, which was properly appreciated by the members.

The Society adjourned on the 21st, after a session of three days, to meet at Covington, on the third Wednesday of October next.

We append the letter of Dr. SNEED.

*To the Members of the Kentucky State Medical Society.*

GENTLEMEN,

At the last meeting of the Kentucky State Medical Society a resolution was passed making it the duty of the Secretary to present each member of the Society with a statement of the amount of his dues, with the request that the same should be forwarded at as early a day as practicable, in order that the Committee of Publication might be enabled to contract for the printing of the last transactions of the Society.

I was further directed, by the same resolution, to strike from the list all such members as failed to comply with this order. If each member will forward the amount of his dues, the Society will be able to publish, at once, the valuable papers read at the last meeting.

Very respectfully,

W. C. SNEED, *Rec. Sec.*

